# Association between traumatic brain injury and mental health care utilization: evidence from the Canadian Community Health Survey

**DOI:** 10.1186/s40621-023-00424-x

**Published:** 2023-03-13

**Authors:** Nelofar Kureshi, David B. Clarke, Cindy Feng

**Affiliations:** 1 Division of Neurosurgery, Department of Surgery, Dalhousie University, Halifax, NS Canada; 2 Department of Community Health and Epidemiology, Dalhousie University, Halifax, NS Canada

**Keywords:** Traumatic brain injury, Mental health, Health care utilization, Prevalence

## Abstract

**Background:**

Mental health disorders are a common sequelae of traumatic brain injury (TBI) and are associated with worse health outcomes including increased mental health care utilization. The objective of this study was to determine the association between TBI and use of mental health services in a population-based sample.

**Methods:**

Using data from a national Canadian survey, this study evaluated the association between TBI and mental health care utilization, while adjusting for confounding variables. A log-Poisson regression model was used to estimate unadjusted and adjusted prevalence ratios (PR) and 95% confidence intervals (CI).

**Results:**

The study sample included 158,287 TBI patients and 25,339,913 non-injured individuals. Compared with those were not injured, TBI patients reported higher proportions of chronic mental health conditions (27% vs. 12%, *p* < 0.001) and heavy drinking (33% vs. 24%, *p* = 0.005). The adjusted prevalence of mental health care utilization was 60% higher in patients with TBI than those who were not injured (PR = 1.60, 95%; CI 1.05–2.43).

**Conclusions:**

This study suggests that chronic mental health conditions and heavy drinking are more common in individuals with TBI. The prevalence of mental health care utilization is 60% higher in TBI patients compared with those who are not injured after adjusting for sociodemographic factors, mental health conditions, and heavy drinking. Future longitudinal research is required to examine the temporality and direction of the association between TBI and the use of mental health services.

## Background

Traumatic brain injury (TBI) is a major cause of mortality in Canada, contributing to approximately 23% of all injury-related deaths (Public Health Agency of Canada 2020). TBI is also related to many adverse health outcomes (Oddy et al. 2012; Hwang et al. 2008), such as physical and mental health conditions (Mackelprang et al. 2014; Topolovec-Vranic et al. 2012; Silver et al. 2001), cognitive impairment (Andersen et al. 2014), suicidality (Bahraini et al. 2013), substance use (Taylor et al. 2003), victimization (Bushnik et al. 2015), increased mortality (McMillan et al. 2015), increased health care utilization (Bushnik et al. 2015), and incarceration (Farrer and Hedges 2011). In addition to its impact on patients' health, TBI is also immensely burdensome on the health care system.

Individuals who sustain TBI have a higher burden of comorbid illness which results in increased health care utilization (Bushnik et al. 2015; Albrecht et al. 2019). Mental health disorders are commonly observed in TBI patients. In the first-year post-injury, up to 77% receive a psychiatric diagnosis: anxiety, mood and substance-use disorders are common and often present co-morbidly (Alway et al. 2016; Koponen et al. 2002). The high rates of mental health disorders are associated with worse health outcomes, increased mental health care utilization, and poorer quality of life. Studies using Anderson’s Behavioral Model (Andersen and Newman 2005) have found that a variety of predisposing characteristics, enabling characteristics, and need factors are associated with mental health care utilization. Among predisposing characteristics, military service and older age are significantly associated with mental health service utilization (Coxe et al. 2021). Notably, veterans are much more likely to utilize services, which may be due to comorbidity of combat-related mental health problems and TBI (Miles et al. 2017). As adults with TBI become older, they are less likely to obtain mental health services which may be due to physical or cognitive problems affecting service access (Coxe et al. 2021; Fasoli et al. 2010). Among enabling resources, TBI patients without health insurance are less likely to utilize services, indicating that lack of insurance continues to be a reason for not seeking mental health treatment in the USA (Albrecht et al. 2017). Need factors including worse self-reported health, medical comorbidities and psychiatric comorbidities are consistently reported as the strongest predictors of mental health care utilization. TBI is associated with higher rates of psychiatric comorbidities including posttraumatic stress disorder (PTSD), anxiety, mood disorders, schizophrenia, and substance use disorders; many of these conditions are significantly associated with increased mental health care utilization (Coxe et al. 2021; Narrow et al. 2000).

The available literature examining the association of TBI with mental health care utilization is largely limited to veterans in the USA (Miles et al. 2017; Fasoli et al. 2010; Drag et al. 2013). To date, no population-based studies in Canada have been conducted to highlight differences in mental health care service use by TBI patients versus those who are not injured. The objective of the study was to determine whether participants with TBI are associated with a higher probability of mental health care utilization using a population-based sample from Canada.

## Methods

### Data source

This study utilized individual-level data from the 2017 to 2018 Canadian Community Health Survey (CCHS). The CCHS is a cross-sectional population-representative survey which collects sociodemographic measures, health status, health care utilization, and other health determinants. Individuals who are full-time members of the Canadian Forces, reside in prisons or care facilities, or live on First Nations Reserves, Crown Lands, or in some remote regions of Quebec are excluded from the survey. The CCHS captures approximately 98% of the Canadian population that are 12 years of age or older. Person-level survey weights enable representative estimates across provinces and sociodemographic strata. Detailed methodology and sampling characteristics of the CCHS are described elsewhere (Statistics Canada. Canadian Community Health Survey - Annual Component (CCHS) 2021).

### Study setting and participants

#### Exposure and outcome variables

All respondents who reported that their most serious injury in the past 12 months was a concussion or other brain injury were selected to form the TBI group. Nonrespondents (i.e., those in the categories of “don’t know” and “refusal”) were excluded. Patients with TBI were compared to non-injured respondents, which were defined as those respondents who did not experience an injury in the 12 months prior to the survey interview.

The outcome variable of interest was mental health care utilization, measured based on self-reporting on health professional consults for mental health issued in the past 12 months in patients with and without TBI. Mental health care utilization was determined by asking respondents if they had seen or talked on the telephone to any of the following people in the past 12 months about problems with their emotions, mental health or use of alcohol or drugs: psychiatrist, psychologist, family doctor, nurse, social worker, or other health professional.

#### Control variables

Guided by the current literature, the following variables were identified as important covariates to consider as potential confounders: age, sex, level of education, race, household income, mental health comorbidities, and heavy drinking.

The following variables were recoded to reduce the total number of categories compared owing to the difficulties in making comparisons. Any differences in model Akaike information criterion (AIC) were examined to ensure that reduction of category levels did not impact model fit.

##### Age

Based on evidence of greater mental health utilization in older adults from previous studies, age was dichotomized at 65 years.

##### Sex

Sex was modeled as male or female.

##### Race

Respondents were divided into white and non-white categories.

##### Education

Respondents were grouped into three categories based on the highest level of education attained: less than secondary school graduation, secondary school graduation, or post-secondary certificate diploma or university degree.

##### Income

Household income from CCHS responses was used to measure socioeconomic status (all respondents are asked: “what is your best estimate of the total income received by all household members, from all sources, before taxes and deductions, in the past 12 months?”). The CCHS classified household income into five groups (no income or less than $20,000; $20,000–$39,999; $40,000–$59,999; $60,000–$79,999; $80,000 or more). To reduce sparsity of data among these categories, income was recoded into low, middle and high categories. We first determined that the median household income after tax was $59,800 in 2017. Using the definition of middle class as those who earn between three-quarters and double the median household income after tax, Canada’s middle class in 2017 included households that earn between $44,850 and $119,600 after tax. Using this definition range, we recoded household income into three groups: low income ($0-$39,999), middle income ($40,000–$79,999), and high income (≥ $80,000).

##### Mental health comorbidities

Questions on professionally diagnosed mood and anxiety disorders included “Do you have a mood disorder such as depression, bipolar disorder, mania or dysthymia?” and “Do you have an anxiety disorder such as a phobia, obsessive compulsive disorder or a panic disorder?” Responses to these questions were combined to produce a binary variable for any mental health comorbidity.

##### Heavy drinking

Substance abuse was initially defined as heavy drinking and use of illicit drugs in the past year. However, owing to the high non-response for illicit drugs in the study cohort, only heavy drinking was included as a covariate. Respondents were asked, “How often in the past 12 months have you had four or more drinks on one occasion?” Heavy alcohol consumption was defined as responses of “once a month,” “2–3 times a month,” “once a week” or “more than once a week.”

#### Statistical analysis

Frequencies and percentages were calculated for all variables. We report the prevalence ratio (PR) instead of the odds ratio (OR) given the cross-sectional study design (Barros and Hirakata 2003). The OR may be equivalent to the PR for rare events, but reporting of PR is preferred for cross-sectional studies (Santos et al. 2008). A log binomial model was initially attempted but did not converge. In the final analysis, a log Poisson regression model was used to estimate unadjusted and adjusted prevalence ratios and 95% confidence intervals (CI) for the association of TBI with mental health care utilization and to assess the impact of sociodemographic factors on this association. PRs were interpreted as the relative prevalence of mental health care utilization, considering the TBI cohort as the exposed group and the non-injured population as the unexposed group. A PR of < 1 indicates that a given factor is associated with a decreased occurrence of mental health care utilization, compared with the non-injured population, while a PR > 1 indicates that a factor is associated with an increased occurrence of mental health care utilization. Multicollinearity among the explanatory variables was checked using Variance Inflation Factors (VIF). Interactions were tested between significant variables to assess additive effects.

As a sensitivity of the adjusted model, we compared finer grained age and income categories in estimating mental health utilization. To carry out this comparison, we developed candidate models for age and income modeled as 2, 3, 4, or 5 levels in addition to sex, education, race, mental health comorbidities, and heavy drinking. Using the model with 5 levels of age and income as a the full model, we used the likelihood ratio test (LRT) to compare each candidate model against the full model. AIC differences were calculated as Δ_*i*_ = AIC_*i*_ − AIC_min_, where AIC_*i*_ is the AIC for the *i*th model and AIC_min_ is the minimum AIC among all the models. Based on the rule outlined by Burnham & Anderson, models having Δ_*i*_ ≤ 2 have substantial support (evidence), those in which 4 ≤ Δ_*i*_ ≤ 7 have considerably less support, and models having Δ_*i*_ > 10 have essentially no support (Burnham et al. 2004).

Statistics Canada produced sampling weights for each of the study participants from the CCHS. To account for nonequal probability of selection in the CCHS due to the complex sampling design, sample weights were applied to obtain population-based estimates. Bootstrap weights provided with the CCHS data were used to produce reliable estimates weighted to be representative of the Canadian population. All analyses employed the survey library (svy) in R to address the stratified, complex sampling design of the data. Statistical significance for all analyses was set at a two-sided alpha level of *p* < 0.05.

## Results

The original CCHS for 2017–18 had 31,274,372 respondents and the study sample included 158,287 TBI patients and 25,339,913 non-injured respondents. The response rate for the 2017–18 CCHS was 58.8%; Statistics Canada handles total non-response by adjusting the weight of persons who respond to the survey to compensate for those who do not respond. Weights of the nonrespondents are redistributed to respondents within response homogeneity groups. In order to create the response homogeneity groups, a scoring method is used to define a response probability based on characteristics available for both respondents and nonrespondents (Statistics Canada. CCHS 2020).

TBI and non-injured groups were significantly different across most covariates (Table 1). With respect to demographics, compared with those who were not injured, a higher proportion of TBI patients were < 65 years of age (89% vs. 79%, *p* < 0.001). Fewer patients with TBI reported attainment of postsecondary education (47% vs. 59%). A significantly higher proportion of TBI patients were White (85 vs. 75%, *p* = 0.004). In line with previous reports, chronic mental health conditions were more common in TBI patients versus the non-injured group (27% vs. 12%, *p* < 0.001). The proportion of TBI patients who reported heavy drinking was also significantly higher than those who were not injured (33% vs. 24%, *p* = 0.005).

**Table 1:** Characteristics of TBI and non-injured patients from 2017 to 18 CCHS (weighted *N* and weighted percent)

Characteristics		TBI *N* = 158,287	Non-injured *N* = 25,339,913	*p*-value
Age	12–64 years	140,578 (88.8)	20,109,743 (79.3)	< 0.001
	≥ 65 years	17,709 (11.2)	5,230,170 (20.6)	
Sex	Male	13,091,629 (48.9)	12,248,284 (48.3)	0.834
	Female	80,762 (51.0)	77,525 (51.6)	
Education	Less than secondary	40,567 (25.9)	4,345,694 (17.5)	
	Secondary	41,921 (26.8)	5,771,030 (23.2)	< 0.001
	Postsecondary	73,837 (47.2)	14,768,861 (59.3)	
Income	$0–$39,999	22,889 (14.4)	5,067,657 (20.0)	0.089
	$40,000–$79,000	45,781 (28.9)	6,973,424 (27.5)	
	≥ $80,000	89,585 (56.6)	13,283,231 (52.4)	
Race	Non-white	21,217 (14.6)	6,090,962 (25.4)	0.004
	White	124,250 (85.4)	17,875,298 (74.6)	
Mental health conditions	No	114,849 (72.7)	22,198,432 (87.9)	< 0.001
	Yes	43,160 (27.3)	3,059,757 (12.1)	
Heavy drinking	No	83,611 (67.3)	14,310,147 (76.0)	0.005
	Yes	40,690 (32.7)	4,519,464 (24.0)	

A greater proportion of TBI patients utilized mental health services compared with non-injured patients (24% vs. 13%, *p* = 0.005, Table 2). Those who were ≥ 65 years of age used less mental health care services than those who were younger (6% vs. 16%, *p* < 0.001) and females reported almost twice the amount of service use than males (18% vs. 10%, *p* < 0.001). Among those who reported chronic mental health conditions, 56% used mental health services, but only 16% of the cohort who reported heavy drinking sought help from mental health professionals.

**Table 2:** Characteristics of study cohort utilizing mental health services (weighted *N* and weighted percent)

Characteristics		Mental health care utilization *N* (%)	*p*-value
Injury	Non-injured	858,468 (12.6)	0.005
	TBI	7189 (23.8)	
Age	12–64 years	10,61,436 (16.1)	< 0.001
	≥ 65 years	108,590 (6.4)	
Sex	Male	400,333 (9.8)	< 0.001
	Female	769,693 (18.3)	
Education	Less than secondary	188,870 (11.8)	< 0.001
	Secondary	213,919 (13.6)	
	Postsecondary	751,015 (15.1)	
Income	$0–$39,999	311,896 (15.7)	0.006
	$40,000–$79,000	318,167 (13.0)	
	≥ $80,000	539,068 (14.0)	
Race	Non-white	102,227 (10.7)	0.001
	White	1,008,564 (14.4)	
Mental health conditions	No	618,807 (8.5)	< 0.001
	Yes	547,493 (55.9)	
Heavy drinking	No	681,453 (14.1)	0.034
	Yes	295,112 (15.9)	

In an unadjusted analysis (Table 3), we observed an increased prevalence of mental health service use by the TBI cohort (PR = 1.88, 95% CI 1.22–2.91). Respondents who were female (PR = 1.86, 95% CI 1.70–2.05), had postsecondary education (PR = 1.28, 95% CI 1.14–1.44), received an income of < CAD $39,999 (PR = 1.12, 95% CI 1.00–1.24), and identified as White (PR = 1.35, 95% CI 1.12–1.63) reported significantly greater use of mental health care services. Those who were > 65 years of age had a 40% lower prevalence of mental health service use (PR = 0.40, 95% CI 0.35–0.45). Individuals who reported chronic mental health conditions had a six times higher prevalence of mental health care utilization (PR = 6.58, 95% CI 6.10–7.10) than those who did not. Heavy drinkers accessed mental health services 12% more than those who did not report heavy drinking (PR = 1.12, 95% CI 1.01–1.25).

**Table 3:** Unadjusted and adjusted prevalence rates of mental health care utilization

Characteristics		PR (95% CI)^a^	*p*-value	aPR (95% CI)^b^	*p*-value
Injury	Non-injured	Referent		Referent	
	TBI	1.88 (1.21–2.92)	0.004	1.60 (1.05–2.43)	0.027
Age	12–64 years	Referent		Referent	
	≥ 65 years	0.40 (0.35–0.45)	< 0.001	0.41 (0.35–0.49)	< 0.001
Sex	Male	Referent		Referent	
	Female	1.86 (1.70–2.05)	< 0.001	1.70 (1.52–1.91)	< 0.001
Education	Less than secondary	Referent		Referent	
	Secondary	1.115 (1.00–1.32)	0.42	1.06 (0.89–1.25)	0.506
	Postsecondary	1.28 (1.14–1.44)	< 0.001	1.27 (1.10–1.46)	0.001
Income	≥ $80,000	Referent		Referent	
	$0–$39,999	1.12 (1.00–1.24)	0.032	1.09 (0.97–1.25)	0.739
	$40,000–$79,999	0.93 (0.84–1.03)	0.177	0.98 (0.87–1.09)	0.149
Race	Non-white	Referent		Referent	
	White	1.35 (1.12–1.63)	0.002	1.03 (0.80–1.33)	0.792
Mental health conditions	No	Referent		Referent	
	Yes	6.58 (6.10–7.10)	< 0.001	5.99 (5.41–6.63)	< 0.001
Heavy drinking	No	Referent		Referent	
	Yes	1.12 (1.01–1.25)	0.034	1.07 (0.95–1.22)	0.250

^a^*PR* Prevalence ratio (unadjusted)

^b^*aPR* Prevalence ratio (adjusted)

The prevalence of mental health care utilization was 60% higher in patients with TBI than those were not injured (adjusted prevalence rate [aPR] = 1.60, 95% CI 1.05–2.43), adjusted for age, sex, race, education, income, mental health conditions, and heavy drinking (Table 3). The prevalence of mental health service use remained significantly higher in females (aPR = 1.70, 95% CI 1.52–1.91) and those with postsecondary education (aPR = 1.25, 95% CI 1.10–1.46) in the adjusted model. Age ≥ 65 years was associated with a 59% lower prevalence of mental health care utilization (aPR = 0.41,9 5% CI 0.35–0.49). Similar to the unadjusted model, the prevalence of mental health care utilization was six times in higher in those with a history of mood disorders or anxiety (aPR = 5.99, 95% CI 5.41–6.63). Heavy drinking was associated with a 7% increase in mental health care service after adjusting for other covariates, but this association was not statistically significant (*p* = 0.24). Model comparisons using LRT confirmed that the selected model was not significantly different from the full model and had Δ_*i*_ = 2 (Table 4).

**Table 4:** Model comparison using likelihood ratio tests and Akaike information criterion

Model^a^	LRT^b^	*p*-value	AIC^c^
Model 1^d^: age 5 levels, income 5 levels	–	–	32.11163
Model 2^e^: age 4 levels, income 4 levels	1.226908e−05	0.99999	28.11165
Model 3^f^: age 3 levels, income 3 levels	3.007202e−05	1.0000	24.11168
Model 4^g^: age 2 levels, income 2 levels	0.0004498243	1.0000	20.1123
Model 5^h^: age 2 levels, income 3 levels	0.0004483501	1.0000	22.1123

^a^All models include injury type (TBI/non-injured), sex(male/female), race(white/non-white), education (less than secondary/secondary/ postsecondary), mental health conditions (yes/no), heavy drinking (yes/no)

^b^Likelihood ratio test

^c^Akaike information criterion

^d^Age categorized as 5 levels: 12–17, 18–24, 25–44, 45–64, ≥ 65 years; income categorized as 5 levels: no income or < $20,000, $20,000–$39,000, $40,000–$59,000. $60,000–$79,000, ≥ $80,000

^e^Age categorized as 4 levels:12–24, 25–44, 45–64, ≥ 65 years; income categorized as 4 levels: $0–$39,000, $40,000–$59,000, $60,000–$79,000, ≥ $80,000

^f^Age categorized as 3 levels: 12–44, 45–64, ≥ 65 years; income categorized as 3 levels: $0–$39,000, $40,000–$79,000, ≥ $80,000

^g^Age categorized as 2 levels: 12–64, ≥ 65 years; income categorized as 2 levels: $0–$39,000, ≥ $40,000

^h^Age categorized as 2 levels: 12–64, ≥ 65 years; income categorized as 3 levels: $0–$39,000, $40,000–$79,000, ≥ $80,000

## Discussion

This study examined the association of TBI with mental health care utilization in a representative sample of the Canadian population. Compared with the non-injured group, the TBI cohort was comprised largely of young White individuals who had attained less than postsecondary education. Heavy drinking was significantly more common in patients with TBI and these individuals reported more than twice the prevalence of chronic mental health conditions including mood disorders and anxiety. In this large population-based sample, the prevalence of consultation requests for mental health services was 60% higher in patients with TBI than those who were not injured, after adjusting for age, sex, education, income, mental health conditions, and drinking history.

### Demographics

Among socioeconomic factors, age, sex, and education were significantly associated with mental health service utilization. We observed that individuals ≥ 65 years had a 59% lower prevalence of mental health service use in our study sample. Previous research indicates that there is a disproportionate underutilization of professional mental health services with increasing age, where negative attitudes toward mental health care and lack of perceived need for treatment are the main barriers (Lavingia et al. 2020). Research examining the connection between sex and the utilization of outpatient health services for emotional or psychiatric issues, largely reveals that women are more likely to seek care than men (Albizu-Garcia et al. 2001). In line with this finding, our study found that females had a 70% higher prevalence in seeking mental health treatment than males. Individuals in the study cohort with postsecondary education had a 27% higher prevalence of mental health service use; higher educated patients may have greater health knowledge and be better informed about the reasons why they may require health services.

### Mental health conditions

Other studies report that past mental health care utilization and diagnoses of schizophrenia, depression, or substance abuse (Fasoli et al. 2010) and previous history of mental health disorders (Miles et al. 2017; Fasoli et al. 2010; Narrow et al. 2000) are important predictors of mental health care utilization after TBI. Mental health conditions assessed in this study include mood disorders such as depression, bipolar disorder, mania, and dysthymia, as well as anxiety disorders such as a phobia, obsessive compulsive disorder, and panic disorder. Chronic mental health conditions were twice as commonly reported by TBI patients than non-injured respondents; however, the unadjusted rate of mental health service use was 88% higher in TBI patients, suggesting that service utilization underestimates mental health conditions in this population. According to a recent systematic review (Schnyder et al. 2017), help-seeking is often delayed or completely avoided in the general population. Stigma and personal attitudes toward mental disorders or mental health services are regarded as main reasons for insufficient help-seeking. The adjusted PR suggests that mood/anxiety disorders and heavy drinking cannot fully explain the differences in mental health service use between TBI patients and non-injured patients. This notion is supported by Drag et al. who reported that although psychiatric disorders are more prevalent in veterans with TBI and associated with increased medical and mental health care utilization, they cannot entirely account for the significant differences in mental health service use among veterans with and without TBI (Drag et al. 2013).

### Mental health care utilization

Outpatient mental health service use is commonly observed during the pre-injury and post-injury period in major trauma patients (Evans et al. 2023). In our study, TBI was significantly associated with utilization of mental health services, after adjusting for demographic characteristics and mental health diagnoses. Several studies have reported a similar association of mental health care utilization with TBI, especially in the veteran population (Coxe et al. 2021; Miles et al. 2017; Fasoli et al. 2010; Drag et al. 2013). In a subsample of returning veterans who were newly diagnosed with PTSD, depression, and/or anxiety, PTSD and TBI history, but not depression or anxiety, were associated with a greater number of psychotherapy visits when controlling for demographic and clinical variables (Miles et al. 2017). In another study which examined predictors of outpatient and inpatient health care utilization in veterans with a history of TBI, mental health disorders such as mood disorders, substance use disorders, PTSD, and schizophrenia were associated with inpatient and outpatient mental health care utilization (Drag et al. 2013). Although our study did not control for PTSD, schizophrenia, and other less common psychiatric disorders, we similarly observed that TBI history was related to greater mental health care utilization independent of mood and anxiety diagnoses as well as demographic characteristics.

Given the cross-sectional nature of the data, it is impossible to determine whether the mental health service utilization preceded or followed TBI in our study sample. Head injuries and mental health conditions increase the risk of mental health service utilization and are well-known correlates; however, with regards to how and when one increases the likelihood of the other, particularly when controlling for demographic risk factors, remains unknown. The present results, combined with previous research, suggest that the association between TBI and mental health service utilization is intricate and potentially bidirectional (Sheldrake et al. 2022; Zahniser et al. 2019). In individuals with TBI, an increased prevalence of mental health service use may be attributed to multiple factors including past psychiatric disorders, substance abuse, distress related to the injury, cognitive complaints, emotional lability, and behavioral problems. Conversely, individuals with a history of psychiatric disorders may be at increased risk of TBI, further compounding the need for mental health services. Thus, a prospective investigation of the temporality and direction of the relationship between TBI and mental health service utilization seems warranted.

### Strengths and limitations

Strengths of this study include its large sample size of adults with TBI in Canada, a sample whose data were drawn from a national database. This is in contrast to the majority of previous studies which have been conducted in military personnel and are not generalizable to the larger population (Fasoli et al. 2010; Drag et al. 2013; Graves et al. 2019; Dismuke-Greer et al. 2020; Finn et al. 2018; Kehle-Forbes et al. 2017). There are several limitations to our study that must be acknowledged. The survey did not specifically evaluate substance abuse, PTSD, schizophrenia, neurodevelopmental conditions, psychoses, and personality disorders. The CCHS assesses self-reported measures of TBI which may increase the likelihood of misclassification, potentially resulting in conservative estimates. Given the survey questionnaire design, it was not possible to distinguish between severities of TBI in the cohort. Survey questions assessed respondent habits in the previous 12-month period and, therefore, the prevalence of mental health care utilization reported herein is likely an underestimate of the long-term outcome of TBI patients. Finally, due to the cross-sectional study design of the CCHS, a temporal relationship between TBI and mental health care utilization cannot be established, and this precludes any causal inference.

## Conclusions

This study provides a national picture of mental health care utilization by Canadian TBI patients.

Compared with the non-injured population, TBI patients have a significantly higher prevalence of chronic mental health conditions and heavy drinking. The use of mental health services by community-dwelling individuals with TBI is 60% more compared with non-injured individuals after adjusting for sociodemographic factors, presence of mental health conditions, and heavy drinking. Future longitudinal research is required to examine the timing and direction of the association between TBI and the use of mental health services.

## Data Availability

The public use microdata file (PUMF) from the Canadian Community Health Survey (CCHS) 2017–18 are available through Statistics Canada.See https://www150.statcan.gc.ca/n1/en/catalogue/82M0013X

## Abbreviations

CCHS: Canadian Community Health Survey
PR: Prevalence ratio
PTSD: Posttraumatic stress disorder
TBI: Traumatic brain injury

## References

[CR1] Albizu-Garcia CE, Alegría M, Freeman D, Vera M (2001). Gender and health services use for a mental health problem. Soc Sci Med.

[CR2] Albrecht JS, O’Hara LM, Moser KA, Mullins CD, Rao V (2017). Perception of barriers to the diagnosis and receipt of treatment for neuropsychiatric disturbances after traumatic brain injury. Arch Phys Med Rehabil.

[CR3] Albrecht JS, Barbour L, Abariga SA, Rao V, Perfetto EM (2019). Risk of depression after traumatic brain injury in a large national sample. J Neurotrauma.

[CR4] Alway Y, Gould KR, Johnston L, McKenzie D, Ponsford J (2016). A prospective examination of axis I psychiatric disorders in the first 5 years following moderate to severe traumatic brain injury. Psychol Med.

[CR5] Andersen J, Kot N, Ennis N, Colantonio A, Ouchterlony D, Cusimano MD (2014). Traumatic brain injury and cognitive impairment in men who are homeless. Disabil Rehabil.

[CR6] Andersen R, Newman JF. Societal and individual determinants of medical care utilization in the United States. Milbank Q. 2005;83(4). 4198894

[CR7] Bahraini NH, Simpson GK, Brenner LA, Hoffberg AS, Schneider AL (2013). Suicidal ideation and behaviours after traumatic brain injury: a systematic review. Brain Impairment.

[CR8] Barros AJ, Hirakata VN (2003). Alternatives for logistic regression in cross-sectional studies: an empirical comparison of models that directly estimate the prevalence ratio. BMC Med Res Methodol.

[CR9] Burnham KP, Anderson DR, editors. Model Selection and Multimodel Inference [Internet]. New York, NY: Springer; 2004. 10.1007/b97636. Accessed 7 Feb 2023.

[CR10] Bushnik T, Caplan B, Bogner J, Brenner L, To MJ, O’Brien K (2015). Healthcare utilization, legal incidents, and victimization following traumatic brain injury in homeless and vulnerably housed individuals: a prospective cohort study. J Head Trauma Rehabilit.

[CR11] Public Health Agency of Canada. Injury in review, 2020 edition: Spotlight on traumatic brain injuries across the life course [Internet]. 2020. https://www.canada.ca/en/public-health/services/injury-prevention/canadian-hospitals-injury-reporting-prevention-program/injury-reports/2020-spotlight-traumatic-brain-injuries-life-course.html. Accessed 11 Apr 2022.

[CR12] Coxe KA, Lee G, Kagotho N, Eads R (2021). Mental health service utilization among adults with head injury with loss of consciousness: implications for social work. Health Soc Work.

[CR13] Dismuke-Greer C, Hirsch S, Carlson K, Pogoda T, Nakase-Richardson R, Bhatnagar S (2020). Health services utilization, health care costs, and diagnoses by mild traumatic brain injury Exposure: a chronic effects of neurotrauma consortium study. Arch Phys Med Rehabil.

[CR14] Drag L, Renninger C, King R, Hoblyn J (2013). Predictors of inpatient and outpatient healthcare utilization in veterans with traumatic brain injury. J Head Trauma Rehabilit.

[CR15] Evans CCD, Li W, Jagelaviciute G, Morrison C, Ng R, Brogly SB (2023). Outpatient mental health service use in major trauma survivors: a population-based cohort study from Ontario. Canada J Trauma Acute Care Surg.

[CR16] Farrer TJ, Hedges DW (2011). Prevalence of traumatic brain injury in incarcerated groups compared to the general population: a meta-analysis. Prog Neuropsychopharmacol Biol Psychiatry.

[CR17] Fasoli DR, Glickman ME, Eisen SV (2010). Predisposing characteristics, enabling resources and need as predictors of utilization and clinical outcomes for veterans receiving mental health services. Med Care.

[CR18] Finn JA, Lamberty GJ, Tang X, Saylors ME, Stevens LF, Kretzmer T (2018). Postrehabilitation mental health treatment utilization in veterans with traumatic brain injury: a VA TBI model systems study. J Head Trauma Rehabil.

[CR20] Graves JM, Mackelprang JL, Moore M, Abshire DA, Rivara FP, Jimenez N (2019). Rural-urban disparities in health care costs and health service utilization following pediatric mild traumatic brain injury. Health Serv Res.

[CR21] Hwang SW, Colantonio A, Chiu S, Tolomiczenko G, Kiss A, Cowan L (2008). The effect of traumatic brain injury on the health of homeless people. Can Med Assoc J.

[CR22] Kehle-Forbes SM, Campbell EH, Taylor BC, Scholten J, Sayer N (2017). Does co-occurring traumatic brain injury affect VHA outpatient health service utilization and associated costs among veterans with posttraumatic stress disorder? an examination based on VHA administrative data. J Head Trauma Rehabil.

[CR23] Koponen S, Taiminen T, Portin R, Himanen L, Isoniemi H, Heinonen H (2002). Axis I and II psychiatric disorders after traumatic brain injury: a 30-year follow-up study. AJP.

[CR24] Lavingia R, Jones K, Asghar-Ali AA (2020). A systematic review of barriers faced by older adults in seeking and accessing mental health care. J Psychiatr Pract.

[CR25] Mackelprang JL, Harpin SB, Grubenhoff JA, Rivara FP (2014). Adverse outcomes among homeless adolescents and young adults who report a history of traumatic brain injury. Am J Public Health.

[CR26] McMillan TM, Laurie M, Oddy M, Menzies M, Stewart E, Wainman-Lefley J (2015). Head injury and mortality in the homeless. J Neurotrauma.

[CR27] Miles SR, Harik JM, Hundt NE, Mignogna J, Pastorek NJ, Thompson KE (2017). Delivery of mental health treatment to combat veterans with psychiatric diagnoses and TBI histories. PLoS ONE.

[CR28] Narrow WE, Regier DA, Norquist G, Rae DS, Kennedy C, Arons B (2000). Mental health service use by Americans with severe mental illnesses. Soc Psychiatry Psychiatr Epidemiol.

[CR29] Oddy M, Moir JF, Fortescue D, Chadwick S (2012). The prevalence of traumatic brain injury in the homeless community in a UK city. Brain Inj.

[CR30] Santos CAS, Fiaccone RL, Oliveira NF, Cunha S, Barreto ML, de Carmo MBB (2008). Estimating adjusted prevalence ratio in clustered cross-sectional epidemiological data. BMC Med Res Methodol.

[CR31] Schnyder N, Panczak R, Groth N, Schultze-Lutter F (2017). Association between mental health-related stigma and active help-seeking: systematic review and meta-analysis. Br J Psychiatry.

[CR32] Sheldrake E, Al-Hakeem H, Lam B, Goldstein BI, Wheeler AL, Burke M (2022). Mental health outcomes across the lifespan in individuals with persistent post-concussion symptoms: a scoping review. Front Neurol.

[CR33] Silver JM, Kramer R, Greenwald S, Weissman M (2001). The association between head injuries and psychiatric disorders: findings from the new haven NIMH epidemiologic catchment area study. Brain Inj.

[CR19] Statistics Canada. Canadian Community Health Survey: Annual Component (CCHS) [Internet]. 2021. https://www23.statcan.gc.ca/imdb/p2SV.pl?Function=getSurvey&SDDS=3226. Accessed 10 Feb 2022.

[CR34] Statistics Canada. CCHS 2017–2018 User Guide.pdf [Internet]. V1 ed. Statistics Canada, editor. Canadian Community Health Survey: Annual Component (CCHS) 2017–2018. Abacus Data Network; 2020. https://hdl.handle.net/11272.1/AB2/SEB16A/1SHX8R

[CR35] Taylor LA, Kreutzer JS, Demm SR, Meade MA (2003). Traumatic brain injury and substance abuse: a review and analysis of the literature. Neuropsychol Rehabil.

[CR36] Topolovec-Vranic J, Ennis N, Colantonio A, Cusimano MD, Hwang SW, Kontos P (2012). Traumatic brain injury among people who are homeless: a systematic review. BMC Public Health.

[CR37] Zahniser E, Nelson LD, Dikmen SS, Machamer JE, Stein MB, Yuh E (2019). The temporal relationship of mental health problems and functional limitations following mTBI: a TRACK-TBI and TED study. J Neurotrauma.

